# Supplementary figures and images for: Identification of hub genes associated with COVID-19 and idiopathic pulmonary fibrosis by integrated bioinformatics analysis
Source: PLoS One. 2022 Jan 19;17(1):e0262737. doi: 10.1371/journal.pone.0262737 (PMC8769324; doi:10.1371/journal.pone.0262737)

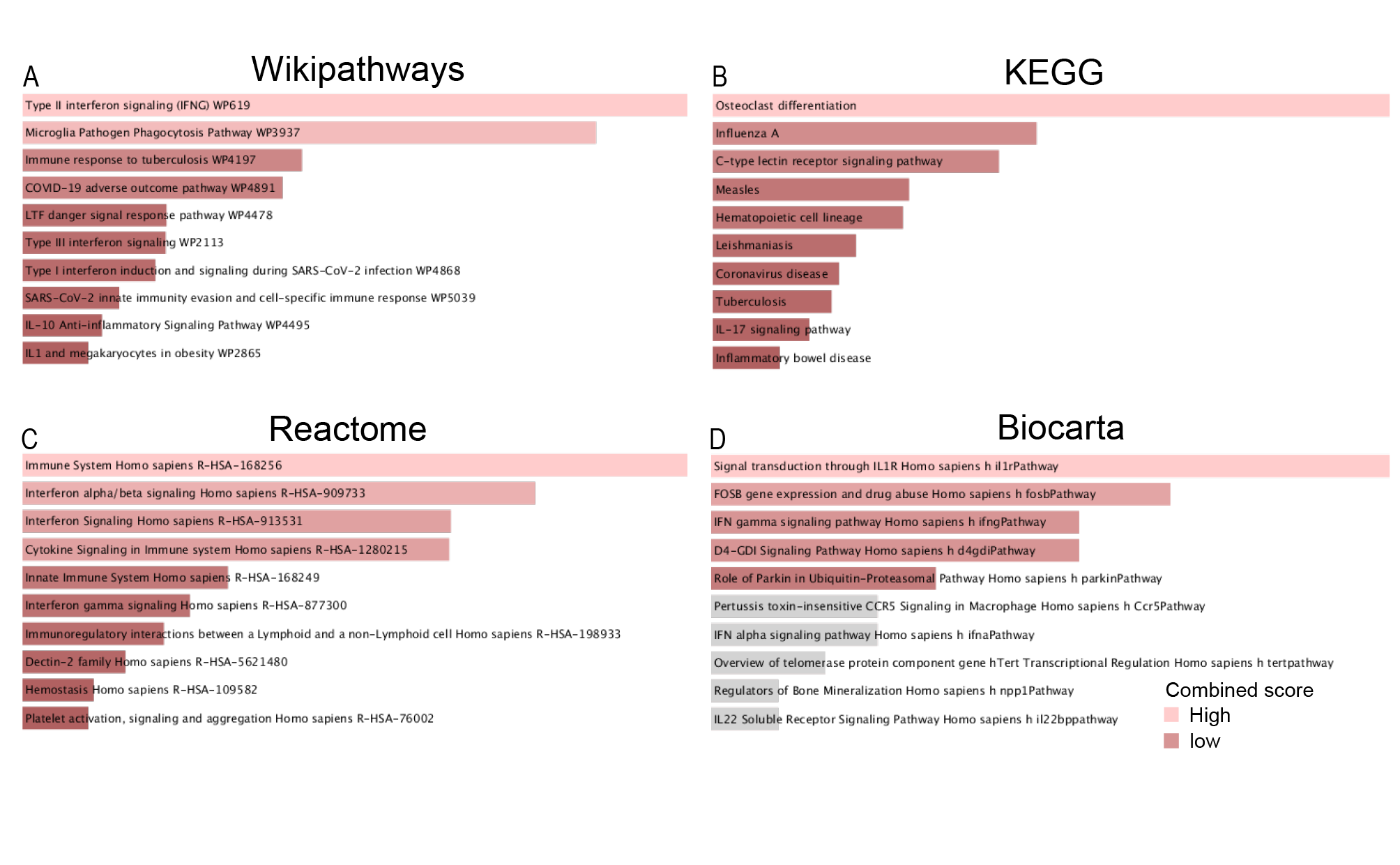

Supplement: S1 Fig — Biological entity of upregulated DEGs between COVID-19 and IPF in Wikipathways (A), KEGG (B), Reactome (C), and Biocarta (D). (TIF) [file pone.0262737.s001.tif]

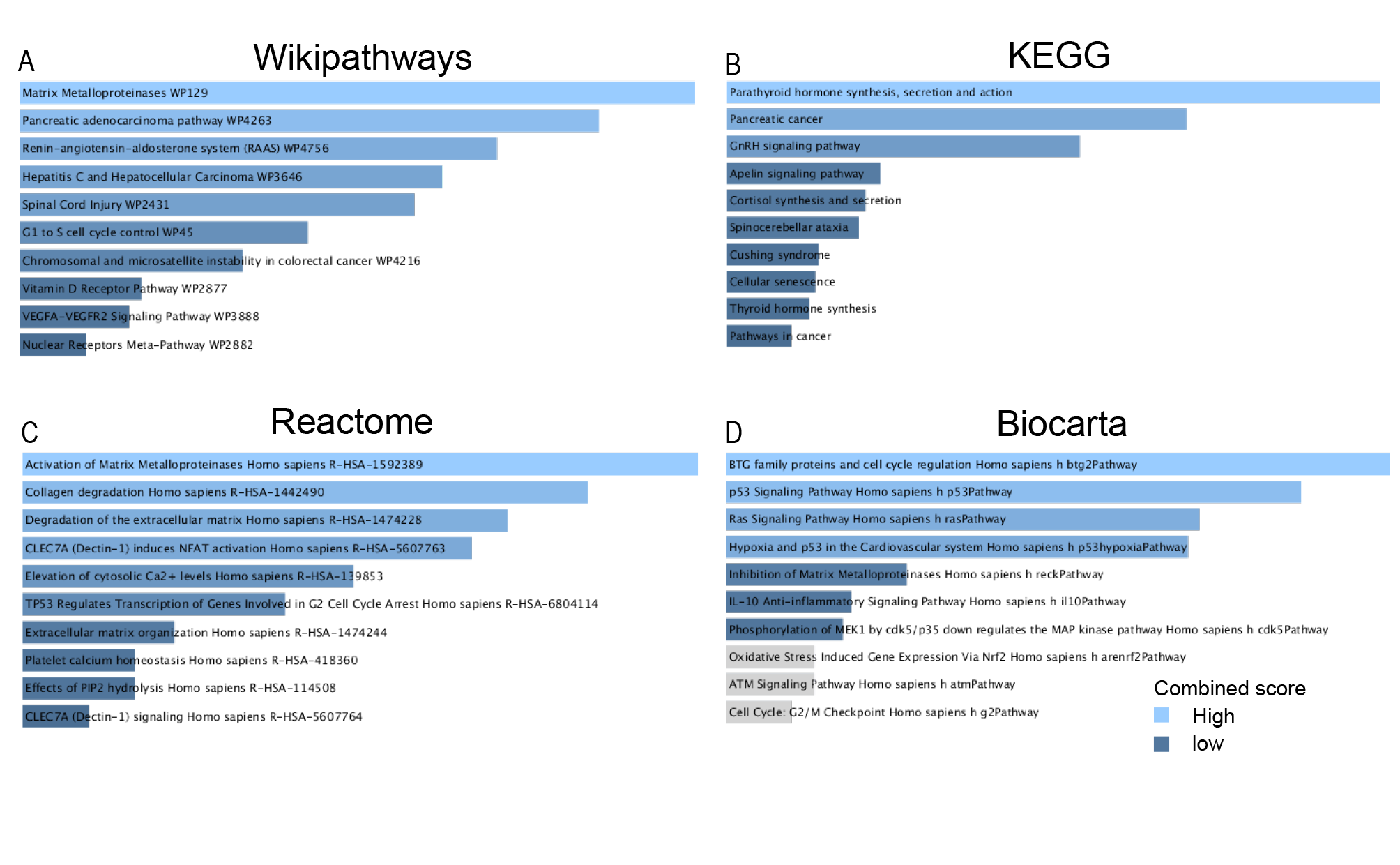

Supplement: S2 Fig — Biological entity of downregulated DEGs between COVID-19 and IPF in Wikipathways (A), KEGG (B), Reactome (C), and Biocarta (D). (TIF) [file pone.0262737.s002.tif]

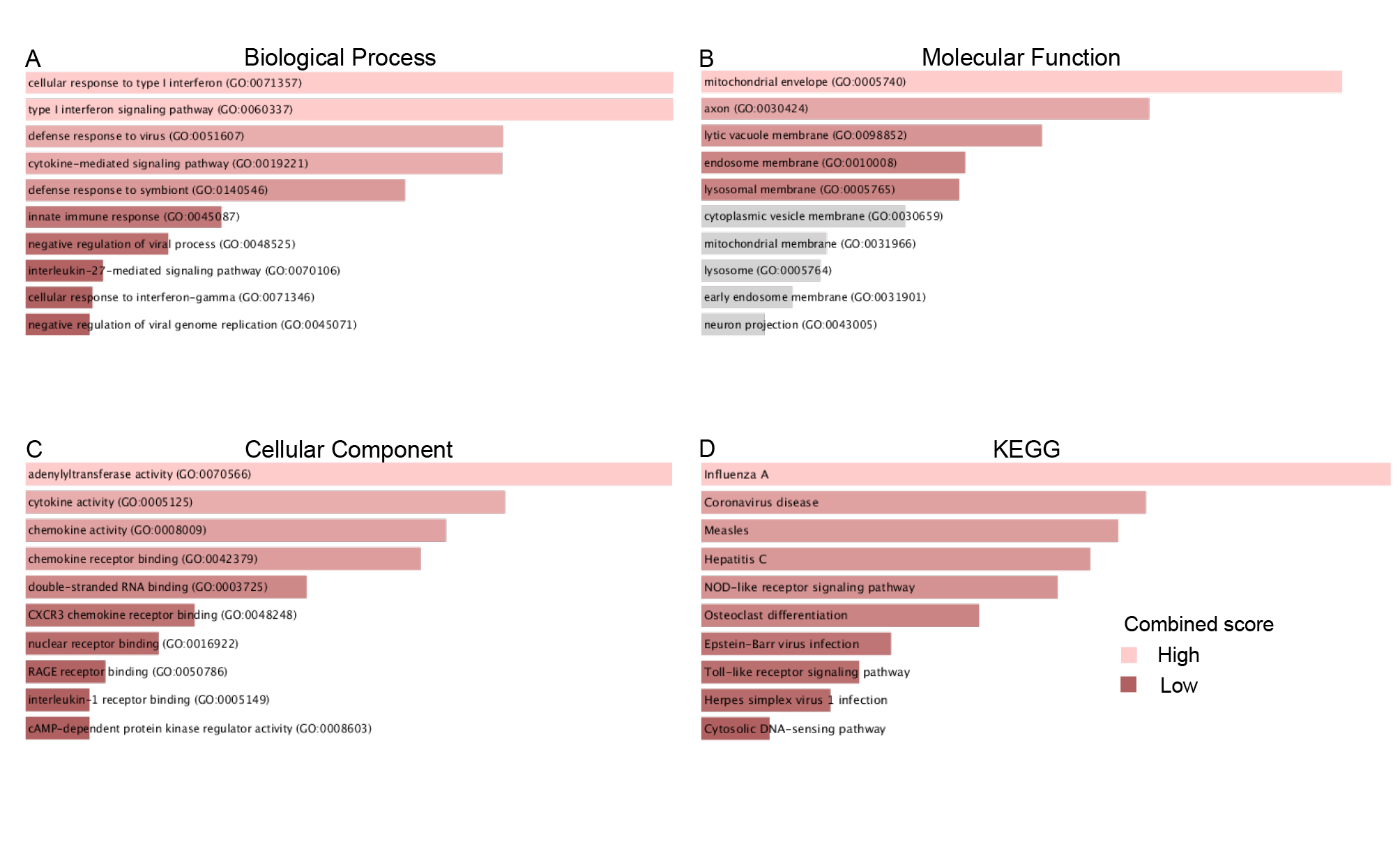

Supplement: S3 Fig — GO analysis of upregulated hub genes according to biological process (A), molecular function (B) and cellular component (C). The results of pathway terms through KEGG analysis of upregulated hub genes(D). (TIF) [file pone.0262737.s003.tif]

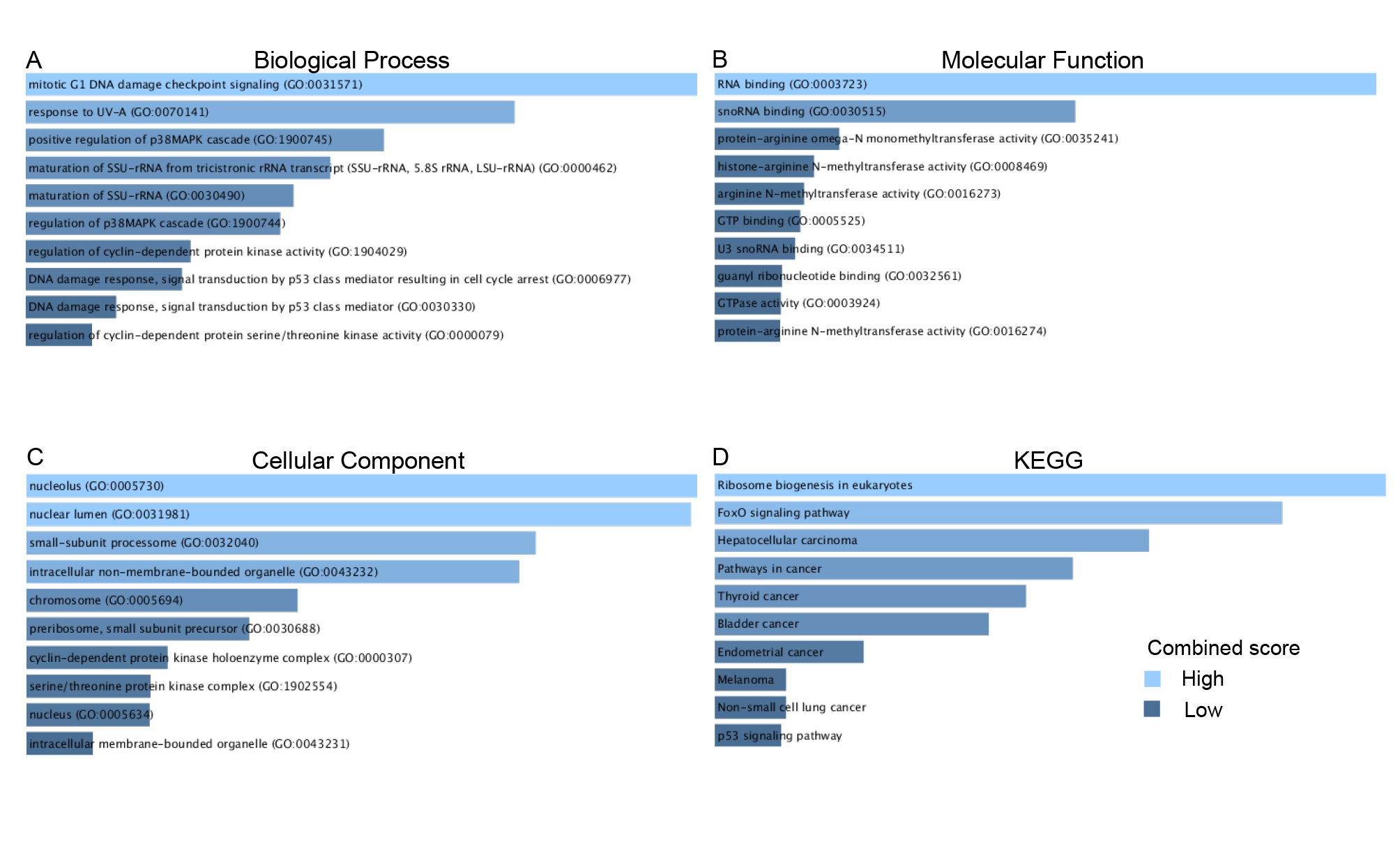

Supplement: S4 Fig — GO analysis of downregulated hub genes according to biological process (A), molecular function (B) and cellular component (C). The results of pathway terms through KEGG analysis of downregulated hub genes(D). (TIF) [file pone.0262737.s004.tif]
