# Supplementary material for: Identification of hub genes associated with COVID-19 and idiopathic pulmonary fibrosis by integrated bioinformatics analysis
Source: PLoS One. 2022 Jan 19;17(1):e0262737. doi: 10.1371/journal.pone.0262737 (PMC8769324; doi:10.1371/journal.pone.0262737)
Supplement: S1 Table — (DOCX) [file pone.0262737.s005.docx]

Supplementary Table 1. Pathway enrichment analysis of high expressed gene between COVID-19 and IPF

| Databases | Pathways | P-value | Genes |
| --- | --- | --- | --- |
| KEGG | Osteoclast differentiation | 8.982948499828465E-12 | IL1A;SPI1;TYROBP;JUND;STAT1;IL1B;NCF4;LCP2;LILRA1;SIRPB1;IRF9;LILRA5 |
|  | Influenza A | 5.8297208963899396E-8 | CXCL10;IL1A;HLA-DRB5;OAS2;STAT1;IL1B;MX1;IRF7;CCL2;IRF9 |
|  | C-type lectin receptor signaling pathway | 1.5206152310511927E-7 | CLEC4D;FCER1G;CLEC7A;STAT1;IL1B;BCL3;CLEC4E;IRF9 |
|  | Measles | 1.4069733605025516E-6 | IL1A;OAS2;STAT1;IL1B;MX1;IRF7;IL2RG;IRF9 |
|  | Hematopoietic cell lineage | 1.6635772474380778E-6 | IL1A;HLA-DRB5;CSF3R;CR1;IL1R2;IL1B;CD37 |
|  | Leishmaniasis | 5.389039525330257E-6 | IL1A;HLA-DRB5;CR1;STAT1;IL1B;NCF4 |
|  | Coronavirus disease | 7.884552614451225E-6 | CXCL10;OAS2;STAT1;IL1B;MX1;CCL2;ISG15;IRF9;C1QC |
|  | Tuberculosis | 9.600115510314953E-6 | IL1A;HLA-DRB5;CR1;FCER1G;CLEC7A;STAT1;IL1B;CLEC4E |
|  | IL-17 signaling pathway | 1.7096839038798434E-5 | CXCL10;JUND;IL1B;CCL2;S100A9;S100A8 |
|  | Inflammatory bowel disease | 3.6126814357117194E-5 | IL1A;HLA-DRB5;STAT1;IL1B;IL2RG |
| WikiPathways | Type II interferon signaling (IFNG) WP619 | 1.542222751552811E-9 | CXCL10;SPI1;STAT1;IL1B;IFI6;ISG15;IRF9 |
|  | Microglia Pathogen Phagocytosis Pathway WP3937 | 2.753389937015107E-9 | FCER1G;TYROBP;NCF4;RAC2;TREM1;SIGLEC7;C1QC |
|  | SARS-CoV-2 innate immunity evasion and cell-specific immune response WP5039 | 2.173927906050966E-6 | CXCL10;IFITM1;STAT1;MX1;IRF7;CCL2 |
|  | Immune response to tuberculosis WP4197 | 8.440950669496818E-6 | IFITM1;STAT1;MX1;IRF9 |
|  | Allograft Rejection WP2328 | 1.2489653851472218E-5 | IL1A;HLA-DRB5;GNLY;STAT1;IL1B;C1QC |
|  | Regulation of toll-like receptor signaling pathway WP1449 | 1.578625588627165E-5 | CXCL10;STAT1;IL1B;IRF7;PELI1;LY96;TREM1 |
|  | TYROBP causal network in microglia WP3945 | 2.6500159760461432E-5 | TYROBP;CD37;SAMSN1;CXCL16;C1QC |
|  | Type I interferon induction and signaling during SARS-CoV-2 infection WP4868 | 2.8948335306473103E-5 | STAT1;OAS2;IRF7;IRF9 |
|  | Glucocorticoid Receptor Pathway WP2880 | 5.174047614570174E-5 | SRGN;MGAM;ALOX5AP;PDE4B;CCL2 |
|  | Non-genomic actions of 1,25 dihydroxyvitamin D3 WP4341 | 5.540662278384389E-5 | OAS2;STAT1;CCL2;ISG15;IFI44L |
| Reactome | Immune System Homo sapiens R-HSA-168256 | 1.0051175819351785E-16 | IFITM3;IFITM1;CSF3R;NCF4;IFI6;LY96;UBE2L6;IL27;ARRB2;LILRA1;  IL2RG;TREM1;SIRPB1;OASL;LILRA5;CLEC7A;S100A12;GBP4;HLA-DRB5;CR1;FCER1G;STAT1;IL1R2;MX1;ISG15;IL1A;CLEC4D;TYROBP;SELL;OAS2;  IL1B;IRF7;PELI1;LCP2;CLEC4E;XAF1;SIGLEC7;IRF9;SIGLEC5;C1QC |
|  | Interferon alpha/beta signaling Homo sapiens R-HSA-909733 | 1.6225586568271503E-13 | IFITM3;IFITM1;OAS2;STAT1;MX1;IRF7;IFI6;ISG15;XAF1;IRF9;OASL |
|  | Cytokine Signaling in Immune system Homo sapiens R-HSA-1280215 | 6.7707686773375905E-12 | IFITM3;IFITM1;HLA-DRB5;CSF3R;STAT1;IL1R2;MX1;IFI6;  UBE2L6;ISG15;IL27;ARRB2;IL2RG;OASL;IL1A;OAS2;IL1B;IRF7;PELI1;XAF1;IRF9;GBP4 |
|  | Interferon Signaling Homo sapiens R-HSA-913531 | 7.070296434632496E-12 | IFITM3;IFITM1;HLA-DRB5;STAT1;MX1;IFI6;UBE2L6;ISG15;OASL;OAS2;IRF7;XAF1;IRF9;GBP4 |
|  | Innate Immune System Homo sapiens R-HSA-168249 | 1.657236573678279E-7 | CR1;FCER1G;LY96;UBE2L6;ISG15;ARRB2;IL2RG;TREM1;SIRPB1;  CLEC4D;TYROBP;CLEC7A;IL1B;IRF7;PELI1;S100A12;LCP2;CLEC4E;C1QC |
|  | Interferon gamma signaling Homo sapiens R-HSA-877300 | 1.089072332032938E-6 | HLA-DRB5;OAS2;STAT1;IRF7;IRF9;GBP4;OASL |
|  | Immunoregulatory interactions between a Lymphoid and a non-Lymphoid cell Homo sapiens R-HSA-198933 | 3.504065031320603E-6 | IFITM1;TYROBP;SELL;LILRA1;TREM1;SIGLEC7;LILRA5;SIGLEC5 |
|  | Dectin-2 family Homo sapiens R-HSA-5621480 | 2.157871492289579E-5 | CLEC4D;FCER1G;CLEC4E |
|  | Hemostasis Homo sapiens R-HSA-109582 | 8.085015327509247E-5 | SRGN;FCER1G;SELL;GNG5;RAC2;IRF7;OLR1;LCP2;ARRB2;PFN1;IL2RG;TREM1 |
|  | Platelet activation, signaling and aggregation Homo sapiens R-HSA-76002 | 1.0827215497504732E-4 | SRGN;FCER1G;GNG5;RAC2;LCP2;ARRB2;PFN1;IL2RG |
| BioCarta | Signal transduction through IL1R Homo sapiens h il1rPathway | 0.018179297107770248 | IL1A;IL1B |
|  | FOSB gene expression and drug abuse Homo sapiens h fosbPathway | 0.028423899449720333 | JUND |
|  | IFN gamma signaling pathway Homo sapiens h ifngPathway | 0.03401184105762086 | STAT1 |
|  | D4-GDI Signaling Pathway Homo sapiens h d4gdiPathway | 0.03401184105762086 | ARHGDIB |
|  | Role of Parkin in Ubiquitin-Proteasomal Pathway Homo sapiens h parkinPathway | 0.04509232682471626 | UBE2L6 |
|  | Pertussis toxin-insensitive CCR5 Signaling in Macrophage Homo sapiens h Ccr5Pathway | 0.05058522944367222 | CCL2 |
|  | IFN alpha signaling pathway Homo sapiens h ifnaPathway | 0.05058522944367222 | STAT1 |
|  | Overview of telomerase protein component gene hTert Transcriptional Regulation Homo sapiens h tertpathway | 0.056046809334145735 | MXD1 |
|  | Regulators of Bone Mineralization Homo sapiens h npp1Pathway | 0.061477243410223426 | ALPL |
|  | IL22 Soluble Receptor Signaling Pathway Homo sapiens h il22bppathway | 0.061477243410223426 | STAT1 |
