# Supplementary material for: Identification of hub genes associated with COVID-19 and idiopathic pulmonary fibrosis by integrated bioinformatics analysis
Source: PLoS One. 2022 Jan 19;17(1):e0262737. doi: 10.1371/journal.pone.0262737 (PMC8769324; doi:10.1371/journal.pone.0262737)
Supplement: S2 Table — (DOCX) [file pone.0262737.s006.docx]

Supplementary Table 1. Pathway enrichment analysis of low expressed gene between COVID-19 and IPF

| Databases | Pathways | P-value | Genes |
| --- | --- | --- | --- |
| KEGG | Parathyroid hormone synthesis, secretion and action | 0.000037499542749716 | EGR1;MMP14;ATF6B;ITPR1;ITPR3 |
|  | Pancreatic cancer | 0.000154245028233339 | RALBP1;CCND1;GADD45A;RALGDS |
|  | GnRH signaling pathway | 0.000335458772073106 | EGR1;MMP14;ITPR1;ITPR3 |
|  | Apelin signaling pathway | 0.00143523254099167 | EGR1;CCND1;ITPR1;ITPR3 |
|  | Cortisol synthesis and secretion | 0.0015994600823125 | ATF6B;ITPR1;ITPR3 |
|  | Spinocerebellar ataxia | 0.00167986450406667 | PSMC5;ITPR1;WIPI1;ITPR3 |
|  | Cushing syndrome | 0.00225349963992794 | ATF6B;CCND1;ITPR1;ITPR3 |
|  | Cellular senescence | 0.00230667499955285 | CCND1;GADD45A;ITPR1;ITPR3 |
|  | Thyroid hormone synthesis | 0.00241082418175346 | ATF6B;ITPR1;ITPR3 |
|  | Pathways in cancer | 0.00274212684919798 | RALBP1;CCND1;MMP1;GADD45A;HMOX1;RALGDS;PGF |
| WikiPathways | Matrix Metalloproteinases WP129 | 1.6247922795874684E-4 | MMP14;MMP1;MMP10 |
|  | Pancreatic adenocarcinoma pathway WP4263 | 2.8353536164372215E-4 | RALBP1;CCND1;GADD45A;RALGDS |
|  | Renin-angiotensin-aldosterone system (RAAS) WP4756 | 5.114902544262766E-4 | ATF6B;ITPR1;ITPR3 |
|  | Hepatitis C and Hepatocellular Carcinoma WP3646 | 7.025701573374969E-4 | CCND1;MMP1;PODXL |
|  | Spinal Cord Injury WP2431 | 8.248755426511761E-4 | EGR1;CCND1;GADD45A;CSPG4 |
|  | G1 to S cell cycle control WP45 | 0.001529507305314004 | CCND1;ATF6B;GADD45A |
|  | Chromosomal and microsatellite instability in colorectal cancer WP4216 | 0.0022318250544541697 | CCND1;GADD45A;RALGDS |
|  | Vitamin D Receptor Pathway WP2877 | 0.0040111917233515365 | S100A2;CCND1;GADD45A;TRAK1 |
|  | VEGFA-VEGFR2 Signaling Pathway WP3888 | 0.004304268717078484 | EGR1;MMP14;CCND1;RAPGEF1;MMP10;PGF |
|  | Nuclear Receptors Meta-Pathway WP2882 | 0.005518325545952257 | EGR1;PSMC5;DNAJC7;CCND1;HMOX1 |
| Reactome | Activation of Matrix Metalloproteinases Homo sapiens R-HSA-1592389 | 0.00019748957670473 | MMP14;MMP1;MMP10 |
|  | Collagen degradation Homo sapiens R-HSA-1442490 | 0.000357464467676696 | MMP14;MMP1;MMP10 |
|  | Degradation of the extracellular matrix Homo sapiens R-HSA-1474228 | 0.000551321410913005 | MMP14;MMP1;A2M;MMP10 |
|  | CLEC7A (Dectin-1) induces NFAT activation Homo sapiens R-HSA-5607763 | 0.000551321410913005 | ITPR1;ITPR3 |
|  | Elevation of cytosolic Ca2+ levels Homo sapiens R-HSA-139853 | 0.0012662395991897 | ITPR1;ITPR3 |
|  | TP53 Regulates Transcription of Genes Involved in G2 Cell Cycle Arrest Homo sapiens R-HSA-6804114 | 0.00183243759471442 | GADD45A;PRMT1 |
|  | Extracellular matrix organization Homo sapiens R-HSA-1474244 | 0.00333063389651785 | MMP14;MFAP1;MMP1;A2M;MMP10 |
|  | Platelet calcium homeostasis Homo sapiens R-HSA-418360 | 0.00411804542046158 | ITPR1;ITPR3 |
|  | Effects of PIP2 hydrolysis Homo sapiens R-HSA-114508 | 0.00411804542046158 | ITPR1;ITPR3 |
|  | CLEC7A (Dectin-1) signaling Homo sapiens R-HSA-5607764 | 0.00527072010177532 | PSMC5;ITPR1;ITPR3 |
| BioCarta | BTG family proteins and cell cycle regulation Homo sapiens h btg2Pathway | 0.000440161889353639 | CCND1;PRMT1 |
|  | BTG family proteins and cell cycle regulation Homo sapiens h btg2Pathway | 0.000944963105654497 | CCND1;GADD45A |
|  | Ras Signaling Pathway Homo sapiens h rasPathway | 0.00226516562913507 | RALBP1;RALGDS |
|  | Hypoxia and p53 in the Cardiovascular system Homo sapiens h p53hypoxiaPathway | 0.00249787275567805 | GADD45A;TAF1 |
|  | Inhibition of Matrix Metalloproteinases Homo sapiens h reckPathway | 0.0280542928380033 | MMP14 |
|  | IL-10 Anti-inflammatory Signaling Pathway Homo sapiens h il10Pathway | 0.0451927319981768 | HMOX1 |
|  | Phosphorylation of MEK1 by cdk5/p35 down regulates the MAP kinase pathway Homo sapiens h cdk5Pathway | 0.0485844938901796 | EGR1 |
|  | Oxidative Stress Induced Gene Expression Via Nrf2 Homo sapiens h arenrf2Pathway | 0.0620331594172908 | HMOX1 |
|  | ATM Signaling Pathway Homo sapiens h atmPathway | 0.0620331594172908 | GADD45A |
|  | Cell Cycle: G2/M Checkpoint Homo sapiens h g2Pathway | 0.075294365950959 | GADD45A |
