# Supplementary material for: Identification of hub genes associated with COVID-19 and idiopathic pulmonary fibrosis by integrated bioinformatics analysis
Source: PLoS One. 2022 Jan 19;17(1):e0262737. doi: 10.1371/journal.pone.0262737 (PMC8769324; doi:10.1371/journal.pone.0262737)
Supplement: S3 Table — (DOCX) [file pone.0262737.s007.docx]

| Supplementary Table 3. Prediction of candidate drugs for high expressed hub genes. | | | |
| --- | --- | --- | --- |
| Name of drugs | P-value | Adjusted P-value | Genes |
| suloctidil HL60 UP | 1.17603903224093E-25 | 1.27835442804589E-22 | IFITM1;STAT1;MX1;IFI6;UBE2L6;ISG15;IFI44L;OASL  ;CXCL10;OAS2;IRF7;CCL2;XAF1;IRF9 |
| prenylamine HL60 UP | 1.06795174564932E-22 | 5.80431773760409E-20 | CXCL10;STAT1;MX1;IFI6;IRF7;ISG15;XAF1;IFI44L;IRF9;OASL |
| acetohexamide PC3 UP | 2.44772613152278E-19 | 8.86892768321756E-17 | IFITM1;STAT1;OAS2;MX1;IFI6;IFI44L;IRF9;OASL |
| chlorophyllin CTD 00000324 | 1.34723424808812E-15 | 3.66110906917947E-13 | CXCL10;IFITM1;STAT1;OAS2;MX1;IFI6;ISG15 |
| 3'-Azido-3'-deoxythymidine CTD 00007047 | 4.95534561362891E-14 | 1.07729213640292E-11 | IFITM1;STAT1;OAS2;IL1B;MX1;IFI6;IRF7;EPSTI1;CCL2;ISG15;IFI44L |
| prochlorperazine MCF7 UP | 1.95377160519666E-13 | 3.53958289141462E-11 | IFITM1;STAT1;IFI6;IRF7;ISG15;IRF9;OASL |
| terfenadine HL60 UP | 2.50627914129244E-13 | 3.89189346654984E-11 | STAT1;MX1;IFI6;IRF7;ISG15;XAF1;IRF9 |
| etoposide HL60 UP | 2.05940731534332E-12 | 2.79821968972273E-10 | STAT1;IL1B;MX1;IFI6;IRF7;CCL2;ISG15;IRF9 |
| Arsenenous acid CTD 00000922 | 8.47115116155285E-11 | 1.02312681251199E-08 | IFITM3;IFITM1;STAT1;MX1;IFI6;UBE2L6;ISG15;IFI44L;CXCL10;OAS2;  IL1B;CCL2;XAF1 |
| propofol MCF7 UP | 3.01735334589948E-09 | 3.27986308699273E-07 | IFITM1;IFI6;ISG15;IRF9;OASL |
| thioridazine HL60 UP | 7.81962423231667E-09 | 7.7272104913893E-07 | CXCL10;MX1;ISG15;IRF9;OASL |
| mefloquine HL60 UP | 2.55995009368074E-08 | 0.0000023188881265258 | CXCL10;STAT1;MX1;ISG15 |
| Decitabine CTD 00000750 | 7.47482495218659E-08 | 6.22523860218308E-06 | IFITM3;CXCL10;IFITM1;SPI1;STAT1;OAS2;MX1;IFI6;IRF7;UBE2L6;  ISG15;XAF1 |
| Tamibarotene CTD 00002527 | 8.01778660814748E-08 | 6.22523860218308E-06 | TYROBP;STAT1;OAS2;IL1B;IRF7;ISG15;IRF9;OASL |
| Acetovanillone CTD 00002374 | 1.44747055383863E-07 | 0.0000104893366134839 | CXCL10;STAT1;IL1B;CCL2 |
| 3,3',4,4',5-Pentachlorobiphenyl CTD 00001077 | 1.65435185959007E-07 | 0.00001123925294609 | CXCL10;STAT1;IL1B;CCL2;XAF1;IFI44L |
| TPEN CTD 00001994 | 2.43123499162826E-07 | 0.0000155456025641172 | STAT1;IL1B;IFI6;CCL2;S100A12;ISG15 |
| estradiol CTD 00005920 | 4.4564937564329E-07 | 0.0000269122706291253 | IFITM1;STAT1;MX1;IFI6;UBE2L6;ISG15;IFI44L;OASL;CXCL10;OAS2;  IL1B;IRF7;CCL2;CMPK2;XAF1;IRF9 |
| Tetradioxin CTD 00006848 | 5.7685016855077E-07 | 0.0000330019017481414 | IFITM3;IFITM1;STAT1;MX1;IFI6;ISG15;IFI44L;OASL;CXCL10;  OAS2;IL1B;IRF7;CCL2;S100A12;IRF9 |
| allopurinol CTD 00005353 | 7.77644034453368E-07 | 0.0000422649532725405 | CXCL10;STAT1;CCL2 |
| testosterone enanthate CTD 00000155 | 1.10559195826089E-06 | 0.0000572275456490284 | STAT1;OAS2;MX1;IFI6;ISG15;IFI44L;IRF9 |
| HC toxin MCF7 UP | 1.19236564647371E-06 | 0.0000589137026234965 | IFITM1;IFI6;IRF7;UBE2L6;ISG15;OASL |
| hycanthone PC3 UP | 1.51666311734841E-06 | 0.0000716788177633794 | IFITM1;IFI6;OASL |
| Garcinol CTD 00002324 | 1.75488820673873E-06 | 0.0000763025392289999 | STAT1;IL1B;CCL2 |
| triprolidine PC3 UP | 1.75488820673873E-06 | 0.0000763025392289999 | IFITM1;MX1;IFI6 |
| clioquinol HL60 UP | 2.01668701634894E-06 | 0.0000843130302604347 | IL1B;MX1;IRF9 |
| Imatinib CTD 00003267 | 4.87793007937977E-06 | 0.000191699774793969 | CXCL10;STAT1;IL1B;CCL2 |
| benzo[a]pyrene CTD 00005488 | 4.93798867914549E-06 | 0.000191699774793969 | IFITM1;SPI1;STAT1;MX1;IFI6;ISG15;TREM1;CXCL10;TYROBP;  OAS2;IL1B;IRF7;CCL2;XAF1;IRF9 |
| 8-azaguanine HL60 UP | 5.17340730758624E-06 | 0.000193913577356767 | IL1B;IRF7;ISG15;TREM1;IRF9;OASL |
| 1-chloro-2,4-dinitrobenzene CTD 00005848 | 5.53895663858823E-06 | 0.000200694862204847 | CXCL10;STAT1;IL1B;CCL2;CMPK2 |
| RUTIN CTD 00006712 | 0.0000119843547500041 | 0.000420225600427566 | CXCL10;IL1B;CCL2 |
| Gadodiamide hydrate CTD 00002623 | 0.0000138544558881424 | 0.000470618548450339 | CXCL10;CCL2;IFI44L |
| tamoxifen CTD 00006827 | 0.0000163332338930341 | 0.000538006825506911 | IFITM1;STAT1;IL1B;MX1;IFI6;CCL2;IFI44L |
| Fulvestrant CTD 00002740 | 0.0000183691700508583 | 0.00058727317192009 | CXCL10;STAT1;IL1B;IFI6;CCL2 |
| clioquinol PC3 UP | 0.0000205952892395228 | 0.000639630840096037 | MX1;IRF9;OASL |
| Retinoic acid CTD 00006918 | 0.0000212754972265423 | 0.000642401819034766 | IFITM3;IFITM1;SPI1;STAT1;IFI6;UBE2L6;ISG15;TYROBP;OAS2;IL1B;  IRF7;CCL2;XAF1;IRF9 |
| ZINC SULFIDE CTD 00001487 | 0.0000232461242149461 | 0.000664961500569642 | IL1B;CCL2;TREM1 |
| budesonide CTD 00007317 | 0.0000232461242149461 | 0.000664961500569642 | STAT1;IL1B;CCL2 |
| Pyrrolidine dithiocarbamate CTD 00001021 | 0.000025161861523908 | 0.000701306242986873 | TYROBP;IL1B;CCL2;UBE2L6 |
| genistein CTD 00007324 | 0.0000295795984148896 | 0.000803825586924626 | IFITM3;STAT1;IL1B;MX1;IRF7;CCL2;UBE2L6;ISG15 |
| benzene CTD 00005481 | 0.0000404088662296658 | 0.00107132774613772 | IFITM1;MX1;IFI6;CCL2;ISG15;IFI44L |
| DL-Mevalonic acid CTD 00006329 | 0.0000482746163558579 | 0.0012493930471147 | STAT1;IL1B;CCL2 |
| beclomethasone CTD 00005468 | 0.0000528686885140545 | 0.00133647126545993 | CXCL10;IL1B;CCL2 |
| chlorprothixene PC3 UP | 0.0000628977186343321 | 0.00152530795115008 | MX1;IRF9;OASL |
| Cortisol succinate CTD 00000336 | 0.0000631452233686786 | 0.00152530795115008 | STAT1;IL1B |
| Sodium dodecyl sulfate CTD 00006753 | 0.0000655856822173281 | 0.00154981818630947 | CXCL10;IL1B;CCL2 |
| ns-398 CTD 00002958 | 0.0000711850366349372 | 0.00164623741377541 | CXCL10;IL1B;CCL2 |
| PERINDOPRIL CTD 00007376 | 0.000075723892077986 | 0.00164623741377541 | IL1B;CCL2 |
| MIGLITOL CTD 00002031 | 0.000075723892077986 | 0.00164623741377541 | IL1B;S100A12 |
| BISOPROLOL CTD 00007160 | 0.000075723892077986 | 0.00164623741377541 | IL1B;CCL2 |
| Roflumilast CTD 00003916 | 0.0000894323646707883 | 0.00180839602196718 | CXCL10;CCL2 |
| Dexbudesonide TTD 00007528 | 0.0000894323646707883 | 0.00180839602196718 | STAT1;IL1B |
| Pulmicort Nebuamp TTD 00010388 | 0.0000894323646707883 | 0.00180839602196718 | STAT1;IL1B |
| Bathocuproine disulfonate CTD 00001350 | 0.0000898375208704951 | 0.00180839602196718 | TYROBP;CCL2;UBE2L6 |
| acetaminophen CTD 00005295 | 0.0000921970413333254 | 0.00182214879871499 | IFITM3;IFITM1;STAT1;MX1;IFI6;UBE2L6;CXCL10;IL1B;IRF7;  CCL2;CMPK2;XAF1;IRF9 |
| PHENCYCLIDINE CTD 00005881 | 0.0000989711282945408 | 0.0019211002938601 | CXCL10;IL1B;IRF7;CCL2 |
| chitosamine CTD 00006030 | 0.000111426166905545 | 0.00212143365233238 | STAT1;IL1B;CCL2 |
| 1-NITROPYRENE CTD 00001569 | 0.00011319517188158 | 0.00212143365233238 | CXCL10;IFITM1;IL1B;OASL |
| pd 168393 CTD 00004570 | 0.000120229631930328 | 0.00214245262144699 | CXCL10;CCL2 |
| rolipram CTD 00007371 | 0.000120229631930328 | 0.00214245262144699 | CXCL10;IL1B |
| TITANIUM CTD 00006899 | 0.000120229631930328 | 0.00214245262144699 | IL1B;CCL2 |
| azacyclonol PC3 UP | 0.000127546543864549 | 0.00223617892227041 | ISG15;IRF9;OASL |
| 1,3-Dimethylthiourea CTD 00001818 | 0.000137313889638796 | 0.00236920949265669 | IL1B;CCL2 |
| betamethasone CTD 00005504 | 0.000155518877333677 | 0.0026127236510834 | IL1B;CCL2 |
| mitoxantrone CTD 00006345 | 0.000157566975915022 | 0.0026127236510834 | IFITM1;IL1B;IRF7;ISG15 |
| SELENIUM CTD 00006731 | 0.000158638234564401 | 0.0026127236510834 | IFITM3;IFITM1;IL1B;MX1;IFI6;CCL2;TREM1;IRF9 |
| ascorbic acid CTD 00005445 | 0.000172274739639938 | 0.00279490614052777 | CXCL10;STAT1;IL1B;CCL2 |
| Ro 41-5253 CTD 00003040 | 0.000174842334458039 | 0.00279490614052777 | STAT1;OAS2 |
| Phenethyl isothiocyanate CTD 00002443 | 0.000179518143231686 | 0.00282806118395425 | CXCL10;STAT1;CCL2 |
| EIPA CTD 00001833 | 0.000195282003579758 | 0.00303245054130282 | IL1B;CCL2 |
| 5-Fluorouracil CTD 00005987 | 0.000213328996383402 | 0.00322877164777016 | IFITM1;OAS2;IL1B;IFI6;ISG15;XAF1;IRF9 |
| aminoguanidine CTD 00000144 | 0.000216835630439026 | 0.00322877164777016 | STAT1;IL1B |
| Lopinavir CTD 00007472 | 0.000216835630439026 | 0.00322877164777016 | IL1B;CCL2 |
| curcumin CTD 00000663 | 0.000228340313833422 | 0.00335413406941797 | STAT1;IL1B;CCL2;XAF1;TREM1 |
| harmaline CTD 00006074 | 0.000239500964068465 | 0.00342549405187397 | IL1B;CCL2 |
| Polydatin CTD 00002437 | 0.000239500964068465 | 0.00342549405187397 | CXCL10;CCL2 |
| ionomycin CTD 00007090 | 0.000243603710970817 | 0.003438925114614 | TYROBP;IL1B;CCL2 |
| Zinc sulfate CTD 00007264 | 0.000247709616477059 | 0.00345205580910978 | STAT1;IL1B;IFI6;TREM1 |
| Allococaine CTD 00005697 | 0.000257727453675884 | 0.00349000911342452 | CXCL10;IL1B;CCL2;IRF9 |
| Sirtinol CTD 00003986 | 0.000263275756486486 | 0.00349000911342452 | IL1B;CCL2 |
| labetalol HL60 UP | 0.000263275756486486 | 0.00349000911342452 | IFI6;S100A12 |
| dimethoate CTD 00005841 | 0.000263275756486486 | 0.00349000911342452 | IL1B;CCL2 |
| Demecolcine CTD 00005762 | 0.000283135395690558 | 0.00370805030259803 | CXCL10;EPSTI1;CCL2;ISG15;IFI44L;IRF9 |
| Chonsurid CTD 00005665 | 0.000288157763042451 | 0.00372889867175172 | IL1B;CCL2 |
| Prolinedithiocarbamate CTD 00002658 | 0.000314144742212557 | 0.00375247620642911 | IL1B;CCL2 |
| fenbendazole CTD 00005958 | 0.000314144742212557 | 0.00375247620642911 | STAT1;CCL2 |
| stavudine CTD 00007231 | 0.000314144742212557 | 0.00375247620642911 | IL1B;CCL2 |
| protriptyline MCF7 UP | 0.000314144742212557 | 0.00375247620642911 | IFI6;IRF9 |
| rescinnamine MCF7 UP | 0.000314144742212557 | 0.00375247620642911 | IFITM1;IFI6 |
| S-1,2-Dichlorovinyl-N-acetylcysteine CTD 00002159 | 0.000314144742212557 | 0.00375247620642911 | OAS2;MX1 |
| 2,6-DICHLOROINDOPHENOL CTD 00007030 | 0.000314144742212557 | 0.00375247620642911 | CXCL10;STAT1 |
| Pomalidomide CTD 00004109 | 0.000341234455643778 | 0.0039063692440593 | SPI1;CCL2 |
| naloxone CTD 00006373 | 0.00034140301581015 | 0.0039063692440593 | CXCL10;STAT1;IL1B;CCL2;CMPK2 |
| clonidine CTD 00005689 | 0.000341234455643778 | 0.0039063692440593 | IL1B;CCL2 |
| NICKEL SULFATE CTD 00001417 | 0.00034140301581015 | 0.0039063692440593 | CXCL10;STAT1;IL1B;CCL2;CMPK2 |
| Dinoprostone CTD 00007049 | 0.000352530200869042 | 0.000352530200869042 | CXCL10;IL1B;CCL2 |
| Antimycin A CTD 00005427 | 0.000369424668157064 | 0.00409759810496662 | IL1B;CCL2 |
| nicotinamide CTD 00006391 | 0.000369424668157064 | 0.00409759810496662 | IL1B;CCL2 |
| Oxazolone CTD 00006449 | 0.000377509166607356 | 0.00414497438487067 | STAT1;CCL2;CMPK2 |
| cycloheximide CTD 00005731 | 0.000392671246240047 | 0.00426833644662931 | IL1B;IFI6;CCL2;S100A12 |
| CP-55940 CTD 00002329 | 0.000398713147884144 | 0.00429110090841648 | IL1B;CCL2 |
| Digoxigenin CTD 00005824 | 0.000429097665836673 | 0.00450301139756068 | IL1B;CCL2 |
| 15442-64-5 CTD 00000915 | 0.000429097665836673 | 0.00450301139756068 | STAT1;IL1B |
| glutathione CTD 00006035 | 0.000430830897282714 | 0.00450301139756068 | STAT1;IL1B;CCL2 |
| indomethacin CTD 00006147 | 0.00044549363881948 | 0.00461191986092167 | CXCL10;STAT1;IL1B;CCL2 |
| benfluorex MCF7 UP | 0.000460575996545202 | 0.00467893559107135 | IRF9;OASL |
| lanatoside C CTD 00000952 | 0.000460575996545202 | 0.00467893559107135 | IL1B;CCL2 |
| cyclosporin A CTD 00007121 | 0.000480170040657987 | 0.00483282253884474 | STAT1;MX1;IFI6;UBE2L6;ISG15;IFI44L;OASL;CXCL10;OAS2;IL1B;  CCL2;XAF1;IRF9 |
| TITANIUM DIOXIDE CTD 00000489 | 0.000519533304169694 | 0.00514437449955966 | CXCL10;IL1B;CCL2 |
| TIRON CTD 00006898 | 0.000526805209192689 | 0.00514437449955966 | IL1B;CCL2 |
| GNF-Pf-78 TTD 00008278 | 0.000526805209192689 | 0.00514437449955966 | STAT1;IL1B |
| ellagic acid CTD 00005891 | 0.000530055146228779 | 0.00514437449955966 | IL1B;CCL2;IRF9 |
| bepridil MCF7 UP | 0.000561551655870558 | 0.00531627299728311 | IRF9;OASL |
| chloroquine CTD 00005645 | 0.000561551655870558 | 0.00531627299728311 | IL1B;CCL2 |
| ibuprofen CTD 00006137 | 0.000562439185545131 | 0.00531627299728311 | CXCL10;IL1B;CCL2 |
| CADMIUM SELENIDE CTD 00002452 | 0.000597383044336062 | 0.00555004589054102 | IL1B;CCL2 |
| Pulmicort Nebuamp BOSS | 0.000597383044336062 | 0.00555004589054102 | STAT1;IL1B |
| thioridazine MCF7 UP | 0.000630956270455313 | 0.00574567515193901 | IFI6;ISG15;OASL |
| POLY I-C CTD 00006579 | 0.000634297164887471 | 0.00574567515193901 | OAS2;MX1 |
| fluticasone CTD 00002635 | 0.000634297164887471 | 0.00574567515193901 | IL1B;CCL2 |
| eugenol CTD 00005949 | 0.000704601626742558 | 0.00632976833280298 | STAT1;IL1B;CCL2 |
| phenylarsine oxide CTD 00001378 | 0.00071136477869478 | 0.00633814356099366 | STAT1;IL1B |
| dioxidanide CTD 00006819 | 0.000751513868037167 | 0.00663265584415719 | IL1B;CCL2 |
| cinnamaldehyde CTD 00000671 | 0.000756623113776902 | 0.00663265584415719 | IL1B;MX1;IFI44L |
| OZONE CTD 00006460 | 0.000792736881896988 | 0.00678507866631516 | STAT1;IL1B |
| clenbuterol HL60 UP | 0.000792736881896988 | 0.00678507866631516 | IL1B;TREM1 |
| usnic acid CTD 00002807 | 0.000792736881896988 | 0.00678507866631516 | CXCL10;IL1B |
| NICKEL CHLORIDE CTD 00001064 | 0.000858447750193786 | 0.00729009925359879 | IL1B;IRF7;CCL2;IFI44L |
| MG-132 CTD 00002789 | 0.000867884817259809 | 0.00731310694853808 | STAT1;IL1B;CCL2 |
| cilostazol CTD 00002032 | 0.000878395909700633 | 0.0073447411834199 | IL1B;CCL2 |
| TETRACHLOROETHYLENE CTD 00006849 | 0.00092282754514372 | 0.00759934501190321 | MX1;ISG15 |
| uric acid CTD 00006967 | 0.00092282754514372 | 0.00759934501190321 | IL1B;CCL2 |
| alprostadil HL60 UP | 0.000941315315859798 | 0.00768298223260585 | IL1B;ISG15;TREM1;OASL |
| 124020-07-1 CTD 00007038 | 0.000968324347623737 | 0.00768298223260585 | IL1B;CCL2 |
| Telmisartan CTD 00003021 | 0.000968324347623737 | 0.00768298223260585 | IL1B;CCL2 |
| rimexolone HL60 DOWN | 0.000968324347623737 | 0.00768298223260585 | IL1B;CCL2 |
| Acteoside CTD 00002463 | 0.000968324347623737 | 0.00768298223260585 | CXCL10;CCL2 |
| 9,12-Octadecadienoic acid CTD 00007296 | 0.00101488413577505 | 0.00799405112744557 | IL1B;CCL2 |
| nelfinavir CTD 00007329 | 0.00106250473134167 | 0.00819062851840005 | IL1B;CCL2 |
| etynodiol HL60 DOWN | 0.00106250473134167 | 0.00819062851840005 | IL1B;CCL2 |
| STYRENE CTD 00001125 | 0.00106250473134167 | 0.00819062851840005 | IL1B;CCL2 |
| methane BOSS | 0.00106998091040736 | 0.00819062851840005 | STAT1;IL1B;ISG15 |
| 22-Hydroxycholesterol CTD 00000121 | 0.00111118395874627 | 0.00833004802177381 | STAT1;CCL2 |
| berberine CTD 00005500 | 0.00111118395874627 | 0.00833004802177381 | IL1B;CCL2 |
| metoprolol HL60 UP | 0.00111118395874627 | 0.00833004802177381 | CCL2;S100A12 |
| NICKEL CTD 00006389 | 0.00112048914402552 | 0.00834227191476536 | STAT1;IL1B;MX1 |
| actinomycin D CTD 00005748 | 0.00113765506907174 | 0.00841245619102714 | STAT1;IL1B;CCL2 |
| Inosinic acid BOSS | 0.00116091964619299 | 0.00116091964619299 | CXCL10;IRF7 |
| folic acid CTD 00005997 | 0.00120798483658667 | 0.00866531817126836 | IFITM3;IFITM1;CCL2 |
| Cardidigin CTD 00005822 | 0.00121170962468518 | 0.00866531817126836 | IL1B;CCL2 |
| AMILORIDE CTD 00005369 | AMILORIDE CTD 00005369 | 0.00866531817126836 | IL1B;CCL2 |
| prednisolone CTD 00006605 | 0.00121170962468518 | 0.00866531817126836 | IL1B;CCL2 |
| Dinoprostone BOSS | 0.00124415811470496 | 0.00883921484107382 | CXCL10;STAT1;IL1B |
| Leptomycin B CTD 00001805 | 0.001370383663162 | 0.00967277299907205 | CXCL10;CCL2 |
| clofibrate CTD 00005684 | 0.00142536917798253 | 0.00999597610623877 | IL1B;CCL2 |
