# Supplementary material for: Identification of hub genes associated with COVID-19 and idiopathic pulmonary fibrosis by integrated bioinformatics analysis
Source: PLoS One. 2022 Jan 19;17(1):e0262737. doi: 10.1371/journal.pone.0262737 (PMC8769324; doi:10.1371/journal.pone.0262737)
Supplement: S4 Table — (DOCX) [file pone.0262737.s008.docx]

| 22 hub genes | Downstream molecules |
| --- | --- |
| MX1 |  |
| CCL2 | CCR2, CCR4 |
| CXCL10 | YYIAP1, TAZ |
| TYROBP | ITGAM, GPX1, PYCARD, CXCL16, ADAP2 |
| STAT1 | IFITM1, IRF1, IRF9, IFI35, PSMB8, IFIT1, OAS1, MX1, GIP2,  GIP3, IFIT3, PSMB8 |
| S100A12 | AGER |
| IRF7 | IFNA1, IFNB1, IFNE, IFNK, IFNG |
| IL1B | IRAK1, IRAK2, IRAK3, TOLLIP, MYD88 |
| TREM1 | MYD88, IRAK1, IRAK4, NFкB |
| SPI1 | PRNP, NCAM1 |
| UBE2L6 | FLT3 |
| IFI44L | IRGC, OASL |
| XAF1 | XIAP |
| IRF9 | ISRE, GAS, IFNA, IFNB |
| EPSTI1 | VCP |
| ISG15 | DDX17, DDX58 |
| OASL | double-stranded RNA of EMCV and HCV |
| IFITM1 | ERK |
| CMPK2 | CMP, UMP, dCMP |
| IFI6 |  |
| OAS2 | RNase L |
| IFITM3 |  |
